# Prognostic relevance of disseminated tumor cells in the bone marrow and biological factors of 265 primary breast carcinomas

**DOI:** 10.1186/bcr1360

**Published:** 2005-11-24

**Authors:** Christian Schindlbeck, Theresa Kampik, Wolfgang Janni, Brigitte Rack, Udo Jeschke, Stan Krajewski, Harald Sommer, Klaus Friese

**Affiliations:** 1 1st Department of Obstetrics and Gynecology Klinikum, Ludwig-Maximilians-University, D-80337 Munich, Germany; 2 Burnham Institute, La Jolla, California 92037-1062, USA

## Abstract

**Introduction:**

The prognostic significance of disseminated tumor cells in the bone marrow (DTC-BM) of breast cancer patients has been demonstrated in many studies. Yet, it is not clear which of the primary tumors' biological factors predict hematogenous dissemination. We therefore examined 'tissue micro arrays' (TMAs) of 265 primary breast carcinomas from patients with known bone marrow (BM) status for HER2, Topoisomerase IIα (Top IIa), Ki 67, and p53.

**Methods:**

BM analysis was performed by cytospin preparation and immunocytochemical staining for cytokeratin (CK). TMAs were examined by immunohistochemistry (IHC) for HER2, Top IIa, Ki 67 and p53, and fluorescence *in situ *hybridization (FISH) for HER2.

**Results:**

HER2 (2+/3+) was positive in 35/167 (21%) cases (FISH 24.3%), Top IIa (>10%) in 87/187 (46%), Ki 67 in 52/184 (28%) and p53 (>5%) in 61/174 cases (34%). Of 265 patients, 68 (25.7%) showed DTC-BM with a median of 2/2 × 10^6 ^cells (1 to 1,500). None of the examined factors significantly predicted BM positivity. Significant correlation was seen between HER2 IHC and Top IIa (p = 0.06), Ki 67 (p = 0.031), and p53 (p < .001). Top IIa correlated with Ki 67 and p53, and Ki 67 also with p53 (p = 0.004). After a median follow-up of 60.5 months (7 to 255), the presence of DTC-BM showed prognostic relevance for overall survival (p = 0.03), whereas HER2 (IHC, p = 0.04; FISH, p = 0.03) and Ki 67 (p = 0.04) correlated with disease free survival, and HER2 with distant disease free survival (IHC, p = 0.06; FISH, p = 0.05).

**Discussion:**

The congruence of the examined factors' expression rates indicates a causal line of suppressor, proliferation, and mitosis markers, and growth factor receptors. Hematogenous tumor cell spread seems to be an independent process. The examination of these factors on DTC-BM is the aim of ongoing research.

## Introduction

In recent years, the view on breast cancer has changed from that of a more locoregional process towards an early generalized disease. The importance of curative local treatment, therefore, is decreasing [1] as more and more patients at all stages receive systemic chemo- or hormone therapy, even when tumor size is small or lymph node status is negative [2]. The determination of new prognostic parameters to better discriminate and stratify patients for individualized therapies is the focus of ongoing research. The presence of disseminated tumor cells in the bone marrow (DTC-BM) seems to indicate hematogenous dissemination and proved to be prognostically significant for the subsequent development of metastases and tumor associated death in many studies [3-6]. With this new information, patient subgroups requiring intensified oncological care and treatment could be identified.

Hematogenous tumor cells appear to spread at all stages of carcinogenesis [7]. Tumorigenesis, dissemination, aggregation, and metastasis are very complex processes, probably involving hundreds of biological factors, and giving rise to the heterogeneity of tumors. The determination of some of these factors has shown prognostic impact.

The first several steps of carcinogenesis are characterized by genetic alterations. An imbalance between tumor promotor and suppressor genes leads to accelerated mitosis and proliferation. The *TP53 *tumor suppressor gene, which is located on chromosome 17, encodes the nuclear protein p53, which regulates proliferation of normal cells [8]. In malignant tumors, heterogenous forms of mutations can be found [9]. Most *TP53 *mutations result in an accumulation of nonfunctional protein in the tumor cell nuclei, preventing apoptosis and leading to privileged growth of the transformed cells. Due to the long half-life of these proteins, they can be detected by immunohistochemical staining; 20% to 40% of breast carcinomas show mutations of the *TP53 *gene [10]. Although there is controversy concerning the most suitable method for determining p53 status, many studies revealed that the *TP53 *gene and associated protein mutations have a negative prognostic impact for overall survival [11-13].

Tumor cells are characterized by accelerated mitosis and proliferation. Among several proliferation markers, Ki 67 is evaluated as the most frequent protein in breast cancer. The fact that the Ki 67 protein is present during all active phases of the cell cycle (G1, S, G2, and mitosis), but is absent from resting cells (G0), makes it an excellent marker for determining the growth fraction within tissues. During interphase, the antigen can be exclusively detected within the nucleus; alternatively, in mitosis, most of the protein is relocated to the surface of the chromosomes. Localization of the Ki 67 protein, as well as different isoforms, could play an important role in cell cycle regulation. Nevertheless, the exact biological function of Ki 67 remains unclear, even 20 years after it was first described [14]. In many studies, expression of this marker was associated with reduced disease free and overall survival [15-17] in breast cancer patients.

Another important factor in mitosis is topoisomerase IIα (Top IIa). This nuclear enzyme is one of five identified topoisomerases interacting with the cell's double-helix DNA, thus playing an important role in DNA replication, transcription, recombination, condensation or segregation [18]. Topoisomerases act as targets of many antimitotic and antiproliferative drugs, called topoisomerase inhibitors, of which anthracyclins are the clinically most important in breast cancer. Top IIa gene amplification or protein expression is also reported to be a negative prognostic factor [19] and predictor of mainly anthracyclin response.

Finally, one of the most prominent biological factors of breast carcinomas is the human epithelial growth factor receptor 2 (HER2 or cerbB2), which belongs to a family of four characterized subtypes of growth factor receptors. HER2 is a 185 kDa transmembranous protein coded by a gene on the long arm of chromosome 17. The extracellular segment binds to different ligands while the intracellular section mediates signal transduction to the nucleus by tyrosine kinase activity. HER2, a marker of aggressive tumors, promotes accelerated mitosis, inhibition of apoptosis, neoangiogenesis, tumor cell migration and invasion [20]. Although the prognostic impact of HER2 is under discussion, most studies discovered a significant correlation between HER2 overexpression or amplification and poor survival [21]. Furthermore, HER2 overexpression predicts a poor response to antihormonal and cyclophosphamide-methotrexat-fluorouracil (CMF) therapy, although a better response was seen with anthracyclin, taxane and, most importantly, trastuzumab based treatment [22].

All of the examined factors, when overexpressed, seem to be associated with tumor aggressiveness and reduced survival. If, by hypothesis, this reduced survival is partly due to hematogenous dissemination, a correlation between these factors of tumor biology and DTC-BM could be assumed. To investigate if these biological factors could predict the presence of DTC-BM, and which of them are correlated with survival data, we examined the expression and amplification of HER2, Top IIa, Ki 67, and p53 on tissue micro arrays (TMAs) of 265 breast cancer patients with known bone marrow (BM) status at the time of primary diagnosis.

## Methods

### Patients

All patients who attend the 1st Department of Obstetrics and Gynecology, Ludwig-Maximilians-University, Munich, Germany, for surgical treatment of breast cancer are offered BM aspiration for screening of hematogenous tumor cell dissemination. In addition, patients who come for further chemo- or radiation therapy after primary surgery from other hospitals are offered a BM examination. During the follow up visits, repeated aspirations are proposed in order to monitor the BM status during the course of the disease. The experimental nature of this method and the potential side effects are explained to patients. Futhermore, a written informed consent must have been completed according to the local ethics committee guidelines.

If the primary surgery was performed at our hospital, then histological examination was performed at our institute's histopathology laboratory. For comparison of results, the original patients' files and pathology reports were taken into account. Histological examination and evaluation was made according to the TNM classification. Hormone receptor status was examined immunohistochemically; estrogen receptor positivity was indicated when the immunoreactive score (IRS) was more than 1. For this study, we evaluated breast cancer patients who were initially diagnosed by bone marrow examination and whose paraffin embedded breast cancer tissues had been archived.

### Bone marrow preparation and immunocytochemistry

The procedure of bone marrow aspiration and preparation used in our laboratory has been described previously [23]. BM aspiration was performed at both anterior iliac crests (2 to 8 ml each side), either under general anesthesia prior to primary surgery of breast cancer or under local anesthesia. Samples were collected in heparinized syringes and processed within hours. After centrifugation at 900 g for 30 minutes with a Ficoll-Hypaque (Pharmacia, Freiburg, Germany) density gradient (1.077 g/mol), mononuclear cells were washed, and 2 × 10^6 ^cells centrifuged onto each glass slide at 150 g for 5 minutes. The cytospin slides were dried overnight and then stained immunocytochemically or frozen at -80°C.

The detection of DTC-BM was achieved by staining with the monoclonal antibody A45-B/B3 (Micomet, Munich, Germany), which is directed against common cytokeratin epitopes, including the cytokeratin heterodimers 8/18 and 8/19. The concentration used to detect cytokeratin-positive cells in bone marrow cytospins was 2.0 μg/ml. The specific reaction of the primary antibody is indicated with the alkaline phosphatase anti-alkaline phosphatase technique, combined with new fuchsin staining. For each patient, 2 × 10^6 ^cells were screened manually by bright field microscopy. Because of the absence of any background staining, we obtained no indeterminate results. Stained cells were classified as DTC-BM only if they matched the criteria defined previously [23,24], taking into account morphological aspects and excluding unspecifically stained mononuclear cells or cell clusters. All positive results were evaluated by two independent observers.

The breast cancer cell line BT-20A served as a positive control for cytokeratin immunostaining. The specificity of the antibody reaction was tested using an unrelated mouse-myeloma antibody (Sigma, Deisenhofen, Germany) for isotype control on the patients' bone marrow samples, with an identical number of cells as negative control. Up to now, bone marrow samples from more than 200 patients without malignant disease have been examined, with a false positive rate of 1% [25].

### Preparation and examination of tissue micro arrays

Paraffin embedded tissue blocks from patients who underwent BM aspiration at primary diagnosis were picked out for TMA preparation. Representative tumor areas were either marked on the blocks or compared with the original hematoxylin and eosin (H&E) stained sections.

TMAs were prepared at The Burnham Institute, La Jolla, CA, USA using the following technique. Small tissue biopsies were retrieved from selected regions of archived tissue blocks, and these cylindrical samples (diameter 1.0 mm) from different tumors subsequently arrayed in a new paraffin block. TMAs were constructed using a custom-built precision Tissue Arrayer from Beecher Instruments (Silver Springs, MD, USA). Five micrometer sections from these TMA blocks can then be used for analysis by fluorescence *in situ *hybridisation (FISH) or immunohistochemistry (IHC). The most important advantages of tissue array technology include increased capacity, negligible damage caused to the original tissue blocks, the precise positioning of tissue specimens and the utility of these tissues in different kinds of molecular analyses. It is possible to retrieve 15 to 50 punched samples from each donor block without significantly damaging it.

All TMAs were examined by an independent pathologist on H&E staining first. After this, analysis was done spot by spot following the rows and columns. Allocation of the results was done according to lists provided with the TMAs. For this study, a median of three cores (one to nine) per case could be evaluated. Results for the individual cases were then aggregated by determining the median of all the single examinations.

### Her2, Top IIa, KI 67 and p53 immunohistochemistry

Antibody staining methods were similar for each of the different antibodies. The antibodies and methods of evaluation are summarized in Table 1. In general, slides were deparaffinated in xylol, dehydrated, and endogenuous peroxidase blocked with H_2_O_2 _in methanol. Afterwards, samples were heated in citrate acid (pressure cooker, pH 6.0, 5 minutes) and washed in PBS. Blocking reagent and then the primary antibody were added, followed by the biotinylated anti-mouse secondary antibody. After another washing step in PBS, slides were treated with avidin-biotin complex for 30 minutes. Staining was done with 3,3'-diaminobenzidin and counterstaining with hemalaun. Fixated TMA slides were evaluated by bright field microscopy. At least 100 cells per view were counted. HER2 expression was scored semi-quantitatively using the 0 to 3+ score, following common criteria [26]. In short, absence of staining is scored 0, 1+ indicates the lowest level of detectable staining (<10%) or inhomogeneous weak staining, 2+ moderate homogeneous membrane staining, and 3+ intense homogeneous membrane staining. All 2+ and 3+ cases were regarded as positive.

As positive nuclear staining was homogenous, evaluation of Top IIa, KI 67 and p53 expression was done according to the percentage of positively stained cells only, regardless of the intensity. For statistical evaluation, the median of all staining results (in%) was determined and dichotomisation was completed according to this value.

### Fluorescence *in situ *hybridization

FISH analysis was done with the PathVision Probe (Vysis Inc., Downers Grove, IL, USA), according to the manufacturer's protocol. TMA slides were deparaffinized, dried, and placed into pretreatment buffer (2 × SSC, pH 7, 80°C, 30 minutes). Next, the TMA slides were digested with protease (37°C, 90 minutes). After fixation and denaturation (70% formamide/2 × SSC, pH 7.0 to 8.0), hybridization with fluorescent-labeled probes for the *HER2 *gene and alpha-satellite DNA for chromosome 17 was done at 73°C for 2 minutes and at 37°C overnight. The slides were washed, counterstained with 4'-6'-diamidino-2'-phenylindole (DAPI II) and embedded. For later fluorescence microscopy, slides were stored at -20°C and kept dark. Fluorescence microscopy was carried out with the Zeiss Axioscope/Zeiss Axiocam and AxioVision software (Carl Zeiss, Oberkochen, Germany). Signals of at least 60 cells in three different areas of the tumors were counted. Amplification was evaluated with a ratio between *HER2 *and centromere chromosome 17 signals equaling 2.0 or higher [26].

### Statistics

A compilation of patient data and the results of BM and TMA examinations were stored in an Excel database (Microsoft Corp., Redmond, WA, USA). Statistical evaluation was performed with the software SPSS 12.0 (SPSS Inc., Chicago, IL, USA). The χ^2 ^test and correlation analysis was used to compare immunocytochemical, IHC and patient characteristics. The dependence of disease free survival (DFS), distant disease free survival (DDFS) and overall survival (OS) on the examined factors was calculated by Kaplan-Meier analysis (log-rank test, univariate), and cox regression analysis (multivariate, inclusion stepwise forward).

## Results

### Patient characteristics and histological parameters

Bone marrow aspirates and TMAs of 265 patients were analyzed in this study. The median age of patients was 57 years (31 to 88), 82 patients (31%) being premenopausal and 183 (69%) postmenopausal. Of the 265 patients, 153 patients (58%) presented with pT1 tumors, 81 with pT2 (30.5%), 5 with pT3 (2%), 12 with pT4 (4.5%), and 14 were not classified (5%). Additionally, 122 (46%) patients had lymph node metastases at the time of primary surgery, 136 (51%) were negative, and data for 7 patients were missing. The tumors of 232 patients were graded, of which 21 (9%) were classified as G1, 128 (55%) as G2, and 83 (36%) as G3. The distribution of estrogen receptor, lymphangiosis and hemangiosis together with other factors is shown in Table 2.

Comparing histological parameters with each other, tumor size correlated significantly with lymph node involvement (p < 0.001), grading (p < 0.001) and lymphangiosis (p < 0.001), and lymph node metastasis correlated with grading (p = 0.001), estrogen receptor negativity (p < 0.001), and lymph- and hemangiosis (p < .001 each). Estrogen receptor negativity correlated with lymph- and hemangiosis (p < 0.001 each), and lymphangiosis correlated with hemangiosis (p < 0.001).

As this was a retrospective study, patients received adjuvant treatment according to former recommendations. Of the 265 patients, 139 had no adjuvant therapy at all, making them a very interesting subgroup for following the 'natural course of the disease'. Seventy patients had adjuvant chemotherapy, regimens consisting of CMF (thirty-one patients), anthracyclin containing combinations (twenty-one patients), anthracyclin/taxane combination (ten patients) and taxanes in other combinations (three patients). For five patients, chemotherapy was not classified. Forty-eight patients received antihormonal treatment. No data about adjuvant therapy were given for eight patients.

### Bone marrow

Of all the 265 patients whose BM status was known, 68 (25.7%) showed DTC-BM with a median of 2/2 × 10^6 ^screened cells (1 to 1,500). BM status did not correlate significantly with any of the examined histological factors (Table 2), yet there was a trend that patients with a tumor size of more than 2 cm (p = 0.06) and HER2 FISH positive tumors (p = 0.06) had a higher risk of hematogenous dissemination. HER2 IHC, Top IIa, Ki 67 or p53 did not predict the detection of DTC-BM.

### HER2 immunohistochemistry and fluorescence *in situ *hybridization

Altogether, 167 tumors could be evaluated for HER2 IHC. Of these, 81 tumors were scored 0 (48.5%), 52 (31.1%) 1+, 19 (11.4%) 2+, and 15 (9.0%) 3+. In summary, 132 (71%) tumors were HER2 negative (0, 1+), and 35 (21%) positive (2+, 3+). HER2 IHC positivity was not correlated with the standard histological markers or DTC-BM, but correlated strongly with hemangiosis (p = 0.01), and expression of Top IIa (p = 0.06), Ki 67 (p = 0.031), and p53 (p < 0.001). Looking at patients with HER2 3+ positive tumors only, there was correlation with estrogen receptor negativity (p = 0.009), Top IIa (p = 0.004) and p53 (p = 0.014), but not to other factors.

In the FISH analysis, 121 tumors (75.7%) showed no HER2 amplification, 39 were positive (24.3%) with an amplification (ratio HER2/centromer enumeration probe 17) ≥ 2. Compared to IHC, 10/72 (13.9%) cases graded 0, 9/46 (19.5%) 1+ tumors, 7/16 (43.7%) 2+ and 10/15 (66.7%) 3+ cases were amplified (Table 3). Correlation between IHC and FISH was <0.001. HER2 amplification did not correlate statistically with the other examined factors, but indicated a trend towards BM positivity (p = 0.06).

### Topoisomerase IIα

Overall, 187 tumors could be evaluated for Top IIa expression. The median expression rate was 10% (0 to 90). Top IIa positivity (>10%, n = 87, 46%) was significantly correlated to estrogen receptor negativity (p = 0.026), and Ki 67 (p = 0.002) and p53 expression (p < 0.001). Also, a trend for positivity was seen in HER2 IHC positive patients (p = 0.06). BM status or other factors were not related to Top IIa.

### Ki 67

Tumors of 184 patients could be evaluated for Ki 67, of which 52 (28.3%) showed positive staining (0% to 15%). Positivity for Ki 67 (>0%) was correlated with estrogen receptor negativity (p = 0.034). Only 27.3% (36/132) of the Ki 67 negative tumors, but 43.6% (24/55) of the Ki 67 positive ones, were estrogen receptor negative. There also was significant correlation between the expression of Ki 67 and p53 (p = 0.004).

### P53

Tumors of 181 patients could be evaluated for p53 expression. Of these, 114 (63%) showed staining, with a median of 5% of the cells stained (0 to 100). Positivity of more than 5% was seen in 61 cases (33.7%). Expression of p53 correlated with HER2, Top IIa and Ki 67 expression as well as estrogen receptor negativity (p = 0.03), but not with other histological parameters or DTC-BM. For all of the correlations between factors see Table 4.

### Disease free survival

**Figure 1:**
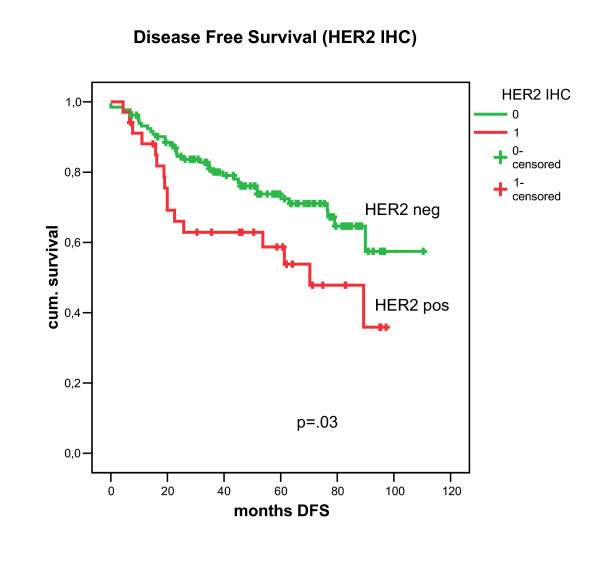
Correlation of disease free survival (DFS) with HER2 immunohistochemistry (IHC) results. Cum, cumulative; neg, negative; pos, positive.

Median observation time of all the patients was 60.5 months (7 to 255). Patients revealing tumor recurrence, metastases, or tumor associated death within six months after BM aspiration have been excluded from follow-up analysis (n = 8). Of the remaining 257 cases, 88 exhibited tumor recurrence (34%), 70 had distant metastases (27%), 18 showed only local recurrence, and 26 showed both local and distant recurrence. As could be expected, tumor size >2 cm (p < 0.001), lymph node involvement (p < 0.001), grading (G3, p = 0.006), lymphangiosis (p < 0.001), and hemangiosis (p < 0.001) all significantly indicated shortened DFS. This was not seen for estrogen receptor negativity (p = 0.52) or the presence of DTC-BM (p = 0.36). Of all the examined stainings, HER2 positivity significantly indicated shortened DFS, both in IHC (p = 0.04; Fig. 1) and FISH (p = 0.03). This was not demonstrated for the HER2 3+ cases only. Also, positive Ki 67 staining was significant for shortened DFS (p = 0.04).

Looking at subgroups of patients in relation to adjuvant therapy, none of the examined factors could significantly predict DFS in the group of patients who underwent chemo- or antihormonal therapy. This was different in the group of patients receiving no adjuvant therapy (n = 138). Again, HER2 IHC (p < 0.001), HER2 FISH (p = 0.003), and Ki 67 positivity (p = 0.008) were significant prognostic factors for reduced DFS.

In multivariate analysis (cox-regression), only tumor size (p = 0.001) and lymphangiosis (p = 0.003) were independent prognostic factors for DFS with a relative risk of 2.2 and 2.1 for tumor recurrence.

### Distant disease free survival

Of the 257 patients, 70 developed distant metastases during the observation period. Again, tumor size (p < 0.001), lymph node involvement (p < 0.001), grading 3 (p = 0.003), lymphangiosis (p < 0.001), and hemangiosis (p < 0.001) may significantly predict the later development of distant metastases. This was not seen for estrogen receptor negativity (p = 0.57). The presence of DTC-BM indicated a trend towards reduced DDFS, but was not significant in this population (p = 0.09). Of the tumor biological factors, HER2 IHC (p = 0.06) and FISH (p = 0.05) also were at the border of significance, whereas Ki 67 positivity again showed a trend towards shortened DDFS (p = 0.09).

In the subgroup of patients receiving chemotherapy, none of the factors was significant for DDFS. In patients receiving hormonal treatment, p53 positivity predicted shortened DDFS (p = 0.023).

In the subgroup not receiving adjuvant treatment, again HER2 IHC (p = 0.003), HER2 FISH (p < 0.001), and Ki 67 (p = 0.02) positivity significantly indicated shortened DDFS.

In multivariate analysis, only tumor size (p = 0.003) and lymphangiosis (p < 0.001) independently predicted reduced DDFS with a relative risk of 2.2 and 2.7 for the development of metastases.

### Overall survival

**Figure 2:**
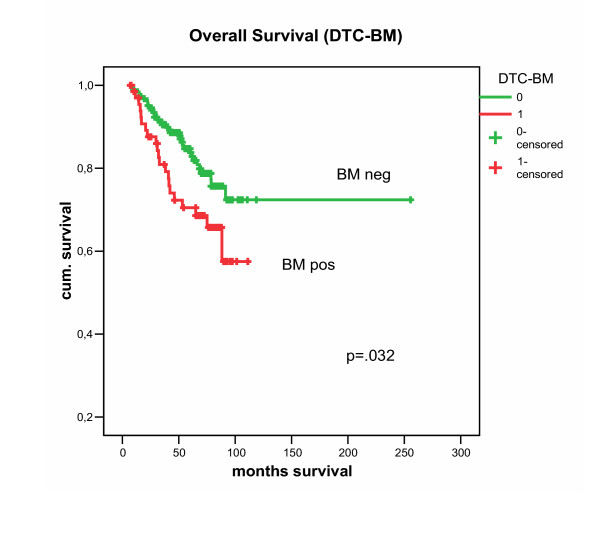
Correlation of overall survival with bone marrow (BM) status (Kaplan-Meier analysis, logrank-test). Cum, cumulative; DTC-BM = DTCs in bone marrow; neg, negative; pos, positive.

During the follow up period, 55 patients (21.4%) died because of tumor associated reasons. As could be expected, tumor size (>2 cm), lymph node involvement, grading 3, lymphangiosis and hemangiosis all predicted reduced OS (p < 0.001 each). For OS, the presence of DTC-BM also was significant (p = 0.03; Fig. 2). Of the 188 patients without presence of DTC-BM, 34 (18%) died because of tumor associated reasons, compared to 21 of 69 patients (30.4%) with positive BM status. Of the 55 patients who died from the disease, 21 (38%) had DTC-BM, compared to only 48 of the 202 patients (24%) who were still alive. Such a correlation was not demonstrated for HER2, Top IIa, Ki 67 or p53. Even for patients with HER2 3+ tumors, OS was not reduced statistically. Looking at subgroups, DTC-BM were also significant in the cohort with hormonal therapy (p = 0.04), whereas HER2 IHC (p = 0.04) and FISH (p = 0.01) were significant in the patients without adjuvant therapy (n = 138). In patients receiving chemotherapy, none of the factors reached significance. The prognostic significance of all the examined factors for OS, DFS, and DDFS is listed in Table 5.

In multivariate analysis, again only tumor size and lymphangiosis remained as independent prognostic factors for OS (p < 0.001 each), with a relative risk of tumor associated death of 3.8 and 2.9, respectively.

## Discussion

Despite the extended use of systemic cytotoxic or hormonal therapy in all stages of breast cancer, the search for factors to discriminate between patients that will benefit most from systemic treatments and for targets of tailored therapies is of major importance. The detection of DTC-BM indicates hematogenous tumor cell spreading and has proved its prognostic significance in many studies [3,5,6,27]. With these promising insights, these cells could be an interesting marker for the status of the disease and targeted therapies. With a prevalence of DTC-BM in 25.7% of all patients and a median of 2 DTCs per 2 × 10^6 ^screened cells (1 to 1,500), our results are in the range of the reported findings [25]. In spite of this, there is little knowledge on the biological behavior of these cells. It is not quite clear which of the primary tumor's factors cause hematogenous spread, which factors are inherited by circulating tumor cells in blood or BM, which factors have to be lost, and which are needed for the later development of distant metastases. As shown, the genetic structure of primary tumors from patients with DTC-BM is different from those of BM negative ones in terms of extracellular matrix modeling, signal transduction, adhesion or angiogenesis factors [28]. To further understand these processes, we looked at the biological factors of tumors from 265 patients with known BM status at the time of primary diagnosis. Staining and hybridization was performed on TMAs of the primary tumors. The tumor TMA technology is a rapid, high throughput survey method to pinpoint molecular markers for detailed studies with conventional tissue specimens. Although TMAs were evaluated by a pathologist for H&E staining, a possible limitation is that the samples acquired from the original tissues, in light of intratumor heterogeneity, may not always be representative of the entire tumor. Methodical problems, mainly the bad quality of older tissue specimens, reduced the number of cases that could be evaluated by up to 30%. Despite differences in IHC staining, evaluation and cutoffs used for statistical analysis, however, expression rates of the investigated factors can be compared to those reported with conventional tissue slides.

Twenty to forty percent of breast carcinomas show mutations of the *TP53 *gene. In our collection, 114 of 181 stained samples (63%) showed expression of a mutated p53 protein, although the intensity of staining was very weak (median 5% of cells, 0 to 100). Using the cutoff of at least 5% stained cells, p53 positivity (33.7%) correlated significantly with Ki 67 (p = 0.004), Her2 (p < 0.001), Top IIa (p < 0.001) and estrogen receptor negativity (p = 0.03), which is in accordance with reported findings [29,30]. There was no correlation with the presence of DTC-BM, even if we used a cutoff of 10% of the stained cells, as suggested by others [31]. p53 could not statistically predict DFS, DDFS or OS; the only significance was found for DDFS (p = 0.023) in patients receiving hormonal therapy. This is in contrast to reported findings, where p53 was a significant prognostic marker [32,33]. Yet, there is still controversy about the most suitable method for determining p53 status, and molecular methods seem to be superior to IHC [12,34,35].

Staining for Ki 67 was evident in 52 of 184 cases (28.3%), which in median was very weak. This is in accordance with similar studies [36], although higher cutoffs of positive staining have been used by others [37]. Again, there was significant correlation of Ki 67 staining with the examined biological factors and estrogen receptor negativity (p = 0.03), but not with DTC-BM. In a study by Fehm *et al*. [38] examining the correlation between Ki 67 and the presence of DTC-BM, the elevated proliferation index significantly predicted the presence of DTC-BM. In our study, Ki 67 positivity was not related to OS, but indicated a trend towards shortened DFS (p = 0.07) and DDFS (p = 0.09) for all patients. In subgroups of patients, those without adjuvant therapy and Ki 67 positive tumors had significantly reduced DFS (p = 0.008) and DDFS (p = 0.06). This confirms findings published by many others [39].

Top IIa expression occurred in 163 of 181 tumors (90%), with a median of 10% positive cells (0% to 90%). This median, which was used as cutoff, has also been described by others [40]. Again, there was no correlation between Top IIa expression and DTC-BM. Top IIa supports DNA decoiling, chromosome segregation during the anaphase of the cell cycle, and DNA replication [41]. Overexpression of Top IIa has predictive value for the effectiveness of therapies involving topoisomerase inhibitors such as anthracyclines. The dependence of anthracyclin chemosensitivity or resistance on the level of Top IIa expression has been shown *in vitro *as well as in clinical studies [42,43]. In a recently published study, Top IIa positivity showed a trend towards prolonged DFS after anthracyclin therapy and reduction of DTC-BM [44]. In this study, neither DFS, DDFS nor OS were related to Top IIa positivity, not even in the subgroup of patients receiving anthracyclin based chemotherapy. The most obvious reason is probably the heterogeneity of the collective, which consisted of small subgroups.

Regarding therapeutic consequences, the most prominent tumor biological factor now is HER2. With a positivity rate of 21% in IHC and 24.3% in FISH, we are within the range of reported findings [45,46]. As expected, there was strong correlation between HER2 amplification and protein expression (p < 0.001), but 66.7% of FISH positive cases in the IHC 3+ group was rather low compared to other studies [47]. Discrepancies between both methods might be due, in part, to bad tissue quality and problems with FISH analysis of older paraffin embedded tissue specimens. There was no relation of HER2 IHC positivity with the detection of DTC-BM, which confirms our former results in a different patient population [48]. Yet, HER2 amplification in FISH showed a trend towards BM positivity (p = 0.06), as was seen in a study by Naume *et al*. [49] showing a more frequent detection of DTC-BM in HER2 positive patients. Despite intensive research in this field, the prognostic impact of HER2 is still under discussion [50]. In our collective, HER2 positivity was a significant prognostic factor for DDFS and DFS, especially in patients receiving no adjuvant therapy. In this subgroup, HER2 positivity also predicted reduced OS. HER2 seems, however, to be rather a predictive factor for the effectiveness of different chemotherapies, including trastuzumab [22].

## Conclusion

Tumorigenesis and dissemination are complex multistep processes. The prognostic value of individual biological factors could, therefore, be more effective in combination as opposed to single factors, which has been demonstrated in several studies [51,52]. For this reason, we analyzed the correlations of different factors with each other. The congruence of the expression rates of the examined tumor biological factors indicates a causal line of suppressor, proliferation and mitosis markers and growth factor receptors. Although targeting of single factors could demonstrate therapeutic efficacy, blocking several steps of tumor cell metabolism might be even more effective. Hematogenous tumor cell spread, however, seems to be rather an independent process. The examination of such different factors on DTC-BM themselves is the aim of ongoing research. The combination of the prognostic impact of DTC-BM and both the prognostic and predictive value of tumor biological factors could help to better stratify patients for individual therapies. DTC-BM comprise a heterogenous 'pool' of cells [7] differing from primary tumors in many ways. As DTC-BM are in a dormant state [53], antimitotic chemotherapy in general has little effect on them [54]. In contrast, antibody based therapy against them is under investigation and has shown promising results. Therapy against the epithelial platelet cell adhesion molecule (EPCAM) surface antigen showed good results in reducing DTC-BM [55]. By determining the HER2 status of circulating tumor cells, Meng *et al*. [56] demonstrated astonishing results with trastuzumab therapy. All these studies point to the potential of using DTC status for the stratification of patients and targeting for tailored therapies. This should be the basis of further research on the detection, enrichment and characterization of DTCs.

## Abbreviations

BM = bone marrow; CMF = cyclophosphamide-methotrexat-fluorouracil; DDFS = distant disease free survival; DFS = disease free survival; DTC = disseminated tumor cell; DTC-BM = DTCs in bone marrow; FISH = fluorescence *in situ *hybridization; HER2 = human epithelial growth factor receptor 2; IHC = immunohistochemistry; OS = overall survival; PBS = phosphate-buffered saline; SSC = sodium chloride-sodium citrate buffer; TMA = tissue micro array; Top IIa = topoisomerase IIα.

## Competing interests

The authors declare that they have no competing interests.

## Authors' contributions

Mrs T Kampik performed IHC staining, and helped to evaluate the results and the follow-up analysis. This study contains material analyzed by Mrs T Kampik in preparation of her thesis to achieve the degree of MD at Ludwig-Maximilians University, Medical Faculty, in Munich, Germany. Mr W Janni and Mrs B Rack carried out BM aspirations and evaluation, organized TMA processing and reviewed the manuscript. Mr U Jeschke gave advice on IHC staining and evaluation and helped to draft the manuscript. Mr S Krajewski performed the TMA preparation, histological examination and manuscript editing. Mr H Sommer and Mr K Friese helped to design and coordinate the study and reviewed the manuscript. All authors read and approved the final manuscript

## Figures and Tables

**Table 1:** Antibodies used for immunohistochemical staining

Factor	Antibody	Company^a^	Dilution	Scoring	Cutoff
HER2	NCL-CB 11	Novocastra	1:40	0, 1, 2, 3	≥ 2+
Top IIa	Ab1, clone SWT3D1	Oncogene	1:40	% stained cells	>10%
Ki 67	Ki 67, clone MIB-1	Dako	1:100	% stained cells	>0%
p53	p53, clone DO-7	Dako	1:200	% stained cells	>5%

HER2, human epithelial growth factor receptor 2; Top IIa, topoisomerase IIα.

^a^Novocastra, Newcastle upon Tyne, UK; Oncogene, Cambridge, MA, USA; DAKO, Hamburg, Germany

**Table 2:** Correlation of characteristics and histological parameters with bone marrow status

Characteristic or histological parameter		No. of patients	Patients (%)	Correlation with BM status (p)
Menopause	No	82	31	0.75
	Yes	183	69	
Tumor size	<2 cm	153	61	0.06
	≥2 cm	98	39	
	na	14		
Lymph node involvement	No	136	53	0.29
	Yes	122	47	
	na	7		
Grading	1, 2	149	64	0.65
	3	83	36	
	na	33		
Estrogen receptor	Positive	141	60	0.85
	Negative	94	40	
	na	30		
Lymphangiosis	No	196	75	0.38
	Yes	64	25	
	na	5		
Hemangiosis	No	245	95	0.58
	Yes	12	5	
	na	8		

BM, bone marrow; na, not available.

**Table 3:** Distribution of HER2 immunohistochemistry and fluorescence *in situ *hybridization

HER2 IHC	HER2 amplification	No. of cases	% FISH positive
0	Negative	62	
	Positive	10	13.9
1+	Negative	37	
	Positive	9	19.5
2+	Negative	9	
	Positive	7	43.7
3+	Negative	5	
	Positive	10	66.7

Correlation p < 0.001. FISH = fluorescence *in situ *hybridization; HER2 = human epithelial growth factor receptor 2; IHC = immunohistochemistry.

**Table 4:** Correlation of histological and tumor biological factors

	p-value											
	Tumor size	LN pos	G = 3	ER	LA	HA	BM	HER2	HER2F	Top IIa	Ki67	p53

Tumor size	-	<0.001	<0.001	0.50	<0.001	0.053	0.063	0.50	0.31	0.09	0.61	0.44
LN pos	<0.001	-	0.001	<0.001	<0.001	<0.001	0.29	0.47	0.17	0.16	0.17	0.45
G = 3	<0.001	0.001	-	0.10	0.001	0.90	0.65	0.11	0.8	0.71	0.80	0.17
ER neg	0.50	<0.001	0.10	-	<0.001	<0.001	0.85	0.29	0.12	0.026	0.034	0.03
LA	<0.001	<0.001	0.001	<0.001	-	<0.001	0.38	0.64	0.57	0.441	0.36	0.98
HA	0.053	<0.001	0.90	<0.001	<0.001	-	0.58	0.01	0.66	0.89	0.87	0.39
BM	0.063	0.29	0.65	0.85	0.38	0.58	-	0.82	0.063	0.47	0.86	0.67
HER2	0.50	0.47	0.11	0.29	0.64	0.01	0.82	-	<0.001	0.06	0.031	<0.001
HER2F	0.31	0.17	0.80	0.12	0.57	0.66	0.063	<0.001	-	0.45	0.55	0.35
Top IIa	0.09	0.16	0.71	0.026	0.441	0.89	0.47	0.06	0.45	-	0.002	<0.001
Ki67	0.61	0.17	0.80	0.034	0.36	0.87	0.86	0.031	0.55	0.002	-	0.004
P53	0.44	0.45	0.17	0.03	0.98	0.39	0.67	<0.001	0.35	<0.001	0.004	-

BM, bone marrow; ER, estrogen receptor; HA, hemangiosis; HER2 = human epithelial growth factor receptor 2; HER2F, HER2 FISH; LA, lymphangiosis; LN, lymph node; Top IIa = topoisomerase IIα.

**Table 5:** Prognostic significance of factors for overall survival, disease free survival, and distant disease free survival

	BM	HER2	HER2F	Top IIa	Ki 67	p53
OS	0.03	0.33^a^	0.22^a^	0.52	0.26	0.98
DFS	0.36	0.03	0.03	0.58	0.04^a^	0.23
DDFS	0.09	0.06^a^	0.05^a^	0.49	0.09^a^	0.22

^a^Significance in the subgroup of patients without adjuvant therapy (n = 138). BM, bone marrow; DDFS, distant disease free survival; DFS, disease free survival; HER2, human epithelial growth factor receptor 2; HER2F, HER2 FISH, OS, overall survival; Top IIa, topoisomerase IIα.
